# Modelling the Plant Uptake of Metals from Release Rates Obtained by the EUF Method

**DOI:** 10.3390/plants11010085

**Published:** 2021-12-28

**Authors:** Manfred Sager

**Affiliations:** Bioforschung Austria, Esslinger Hauptstrasse 134, A-1220 Vienna, Austria; m.sager@bioforschung.at

**Keywords:** EUF, soil dissolution kinetics, lettuce, metal uptake

## Abstract

In this study, soil dissolution kinetics were evaluated to predict the metal uptake of lettuce plants under varying conditions of fertilisation and metal pollution. Velocities and time dependencies of soil dissolution obtained by electro-ultrafiltration (EUF), which prevents back reaction, were modelled in three ways, obtained from suspensions in 0.002 M DTPA at determined soil pH levels, for cases in which sampling versus time led to decreasing concentrations. The models yielded a maximum achievable concentration, a timespan needed for it to be reached, a slope, and an intercept of the respective fitted curves. Three geogenically metalliferous soil samples and one ambient soil sample, both as originals, fertilised with PK or soaked with a Cd-Ni-Pb solution, were used as solid samples. The resulting kinetic parameters were correlated with the amounts absorbed by lettuce plants grown with these substrates in pot experiments, which yielded fairly good correlations with Zn, but also with Li and Sr, as well as Ni and Pb, mainly because of differences due to the addition of a metallic salt solution. Plant growth was hardly influenced by the additions.

## 1. Introduction

The uptake of several elements by healthy green plants from soil depends on the release rate and transport process in the soil, as well as on plant need, the number of receptors, and excretion of plant roots [1,2]. Though some legal thresholds to use arable soils for crop production refer to aqua regia digests (e.g., for Pb and Cd), it has been known for a long time that the total contents present in the soil are not entirely available to plants, due to various release rates, transport processes to the roots, and plant needs. This might be particularly problematic for soils developed on metalliferous rocks [3]. In agriculture, the plant-available “fraction” of a given substrate assumes how much a root can take within one growing season. For reasons of simplicity and costs of investigation, this study sought to imitate this kinetic process by a single-step, partial extraction with organic acids and/or complexants, which release more easily mobilizable elements to the soil solution, irrespective of the chemical speciation of the solid phase of the soil. In sequential leaching, steps of decreasing mobility and plant availability due to dissolution and desorption reactions were obtained, which could be assigned to different solid soil phases, such as exchangeable, carbonate, paedogenic oxide, humic, sulphide, or silicate phases. The availability from these phases varies due to different environmental conditions and time scales. Uncertainties derive from desorption from non-dissolved phases, resorptions at the remaining solid, deviations of chemical properties of the target ion (e.g., no carbonates formed, oxides not easily soluble in acid), or the presence of phases not taken into account by the model [4,5]. Multi-element methods such as ICP–OES permit the extension of element determinations in a solution to much more dissolved elements than originally verified by test minerals. They were thus operationally defined.

In this study, the kinetic process of plant uptake was modelled by soil dissolution kinetics. Contrary to selective leaching methods, which approach mobilisations in a snapshot, kinetic methods simulate desorption from the solid soil particles. This assumes that plant uptake is faster than dissolution, and the uptake is independent of plant needs and uptake exclusion metabolism.

Modelling of release rates versus time permits interpretations of dissolution mechanisms, which may differ among items released from the same substrate [6]. If the release rate is controlled by transport only, it is proportional to the distance from equilibrium, as in the case of film diffusion, intra-particle diffusion, and pore diffusion. Many dissolution processes of geochemical relevance, however, are governed by surface properties, such as crystallisation, impurities, or sorbed species [7].

## 2. Material and Methods

### 2.1. Soil Samples

As test samples, three arable soils from geogenically metalliferous sites and one ambient soil sample from the Austrian Province of Styria were selected (Table 1). The metalliferous soils had developed above sulfidic ore veins containing As, Cd, Cu, Pb, and Zn, but baryte, quartz, pyromorphite, cerussite, and malachite [3,8] have also been occasionally found.

The soil samples were obtained from at least 25 individual cores at 5–30 cm depth, merged on site, dried at 40°, and sieved minor 2 mm [9]. The soil pH was determined in 0.01 M CaCl_2_ [10], the clay–silt–sand distribution by the pipette method [11], the total organic carbon (C-org) by combustion [12], and the pseudo-total contents by inductively coupled plasma emission spectrometry after digestion with aqua regia (Table 1). The statistical validity of these standard methods was annually tested by ring tests run by the ALVA organisation, in which we participated. With respect to precisions, standard deviations of the parameters clay–silt–sand were within 1.5–2.8% absolute, organic carbon within 0.08–0.21% absolute, and soil pH within 0.08–0.14 units. For As, Cr, Ni, Pb, and Zn in aqua regia, precisions of ±6 to 13% of the value were achieved, and for Cd, ± 13 to 20% (unpublished internal data) was obtained.

For the pot experiments, these soils, as well as the resulting soils after the addition of a Cd-Ni-Pb solution or PK fertiliser, were used, with three replicates each.

### 2.2. Test Plants

As test plants, lettuce (*Lactuca sativa*) was chosen, which is known as a rapidly growing and universally accumulating species [13]. Three lettuce seedlings were randomly planted at Kick–Brauckmann pots containing 8 kg of dried soil (≤20 mm), which were placed randomly in a foliar-covered greenhouse, and watered each 3rd day. Then, 10 days after planting, the samples marked as “PK” in Table 2 received an addition of 225 mg/kg P + 128 mg/kg K from a combination of superphosphate and potassium chloride. At the same time, the samples marked as “metal” received an addition of 0.75 mg/kg Cd + 94 mg/kg Ni + 94 mg/kg Pb with respect to the test substrate, from 15 mL of a mixed solution containing 0.384 g Cd (as Cd (NO_3_)_2_·4H_2_O), 48 g Ni (as NiSO_4_·6H_2_O), and 48 g Pb (as Pb(acetate)_2_·3H_2_O) in 1 L, to test the uptake potential of lettuce for those metals. After 40 days of growth, roots and shoots were harvested separately to obtain yields in terms of wet weight. The samples were dried at 65 °C for 72 h, milled, and analysed for total metal contents by simultaneous multi-element analysis by ICP–OES (PerkinElmer Optima 3000XL) after digestion with nitric acid in closed pressure vessels by microwave-assisted heating.

### 2.3. EUF Procedure

After harvest, soil samples from the pot experiments were examined at the Justus Liebig Laboratory of Südzucker AG Company at Rain (Germany) by using a modified electro-ultrafiltration (EUF) method.

**Figure 1:**
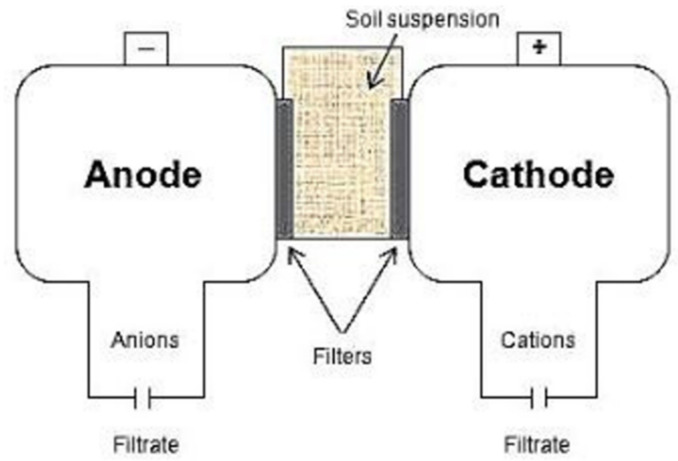
The electro-ultrafiltration (EUF) chamber.

Electro-ultrafiltration (EUF) is a quick method originally designed to determine available plant nutrients and respective fertilisation needs from aqueous soil suspensions, such as nitrate, total dissolved nitrogen, phosphate, and potassium [14,15]. The slurry sample is put into a reaction chamber with electrodes and semipermeable membranes opposite each other, backed by solute-filled chambers for sampling (Figure 1). When the voltage and magnetic stirring are turned on, released ions move towards the respective electrode across the membrane and can be sampled from the anode and cathode chamber without further filtration or centrifugation. The migration velocity is proportional to the electric field strength and inverse to the friction in the fluid; it increases with increasing concentration and temperature. Charged colloids move much slower and hardly penetrate the membranes, which resembles the conditions at the root surface. As liberated ions are rapidly removed from the solid surfaces by the electric field, the back reaction is prevented; thus, the release rate is rapidly experimentally accessible. Variations in EUF conditions are possible within the same reaction cell, to obtain release rates under different conditions. Contrary to release rates obtained by EUF, batch methods target dissolution equilibria.

The experimental EUF parameters had been developed originally to estimate the immediate nutrient availability for ryegrass (*lolium perenne*) and sugar beet at 200 V/20 °C/30 min, which was considered here for practical reasons. Due to H^+^ consumption at the cathode, the cathode space becomes alkaline, and due to OH^−^ consumption at the anode, the anode space becomes acidic. As many hydroxides and hydrated oxides are hardly soluble in the alkaline cathode extract, a complexing agent needs to be added, to determine metal cations (except Na, K). In order to avoid hydroxide precipitations in the cathode chamber, a suspension of 5.00 g of sample in 50 mL 0.002 M DTPA (C_14_H_23_N_3_O_10_) aqueous solution (also known as Merck Titriplex V) was introduced into the EUF chamber system, and five separate fractions were collected every five minutes (in a total of 25 min), applying 200 V and maximum 15 mA at ambient temperature [16]. After each extraction, the released ions were analysed by ICP–OES, and the concentration in solution times ml filtrate yielded the mg/kg soil (listed in Table 3).

### 2.4. Kinetic Modelling

The modelling of release kinetics from soil can be accomplished by using the measured concentrations versus time, but also by using the accumulated (integrated) concentrations. In case the measured concentrations decrease versus time, a time can be extrapolated, when the concentration will reach zero. This means a constant value for the accumulated concentration, such as exhaustion. The corresponding accumulated concentration reached at this time can be regarded as maximum releasable. Although different parameters a and b were obtained from fittings due to different kinetic equations (Table 2), these maximum release concentrations were rather similar.

The integrated curves can be used to model the release mechanism. Fitting parameters are the time to reach the maximum releasable amount, the time to reach it, and the slope and intercept of the fitting curves. The initial release rate is defined just for the linear equation as parameter “a”, but for the Elovich and Weber–Morris equation, it is also “a” for t = 1, which is close to zero with respect to an observation time of 30.

Thus, combining values of plant uptake with parameters “a” would yield correlations to an almost initial release rate in the cases of linear, Elovich, and Weber–Morris equations, and combining values with parameters “b” would yield correlations with the intercept of the accumulated release curve, which means the extrapolated EUF release at zero time in the cases of linear and Weber–Morris equations.

### 2.5. Connections with Plant Uptake

From measured concentrations and yield, the amount of metal content present in lettuce plants after the growth period in the respective substrates was calculated and correlated with the four fitting parameters of the kinetic models.

## 3. Results

Root dry mass reached only about 1/5 of leaf dry mass. Additions of PK fertiliser or metal salt solution hardly affected leaf dry masses but lowered uptake of Pb and increased Cu and Zn. Metal salt additions increased Pb and Ni, as expected. Other authors also found that the addition of P fertiliser lowered the release of Pb from soil [17,18].

The parameters resulting from the EUF procedure, as well as decreasing, increasing, and constant concentrations versus time are discussed in this section. Partition of the release curves into zones of different rate laws was not possible due to the measurement of only five points.

Decreasing concentrations versus time were observed in As, Cd, Cu, Li, Ni, Pb, Sr, and Zn. This means exhaustion of the releasable fraction and possible extrapolation to obtain a maximum releasable amount and the corresponding time. Conversely, the concentrations of Al, Fe, and Co increased versus electrolysis time, which may be interpreted as an activation of release by the dissolution of less soluble coatings [6]. Others, such as Mn, Sb, or V, yielded constant release during the observation period (Table 3).

The linear equation y = b + at (a = slope, b = intercept) would correspond to the dissolution of a homogenous solid. In this case, fitting was worst in most cases.

The Elovich equation y = b + a ln(t) is valid if the release rate decreases because of decreasing surface covering. In this work, this was the case for As, Cd, and Cu, and preferably for Zn. This equation also performed best to model the release and uptake of K from Chinese red clay soils [19].

The Weber–Morris equation y = b + a √t is valid if the transport from reactive surfaces is rate determining; thus, the dissolution is diffusion controlled. The dissolution can be parted in various steps, such as desorption from the solid, diffusion inside the solid, film diffusion, and diffusion within the liquid [20]. The intercept “b” is a diffusion constant and proportional to the interface layer thickness [6,20]. If the intra-particle diffusion is rate controlling, the curves should pass the origin [21]. In this work, the Weber–Morris fit was best in Pb and Sr for all samples, and for Li and Ni in major cases.

The power equation y = b·t^a^ or ln(y) = b + a ln (t) has also been used by some authors to model the release of plant nutrients or dissolution of minerals [19].

In the cases of As and Cd, correlation coefficients between plant uptake and kinetic parameters (Table 4) remained minor 0.45, in spite of additions of soluble Cd. Cu contents correlated for roots only, at best for the Weber–Morris model. Ni, and, to a lesser extent, Pb, correlated positively with the slopes “a”, the intercepts “b”, and the maximum released concentrations, but this was biased by the samples receiving the metal solution.

Zn correlated strongly positively with the slopes “a”, and a little less with the intercept “b”, the maximum release, and the corresponding time of either model. The linear approximation could not fit the curvatures and correlated slightly negatively with its intercept “b”.

Among the cations of low physiological activity for green plants (e.g., Li, Sr), green-plant Li increased with increasing slopes and maximum release, whereas the time to reach this release was not relevant. For Sr, however, linear modelling proved best, and there was a good correlation with the releasable amount and its corresponding time.

Results of all correlation coefficients are presented in Table 4, and the corresponding probability values *p* are listed in Table 5. Correlations with probability values < 0.05 for the null hypothesis (>95% confidence level of significance), calculated as 2-sided *p* from the SPSS statistics program (version 25), are marked *, and those < 0.01 are marked ** in Table 4.

## 4. Discussion

In this work, 0.002 M DTPA (di-ethylenetriaminepenta-acetic acid, C_14_H_23_N_3_O_10_, known as Merck Titriplex V) was used as an electrolyte in the EUF procedure at proper soil pH levels. DTPA is no component of root exudates, but no electrochemical reactions are expectable. Due to its high solubility in water, the free acid can be used, contrary to EDTA, which has to be taken as the Na_2_–salt. DTPA is, therefore, more convenient for the ICP–OES measurement and prevents ion exchanges with Na. Compared with EDTA, the DTPA is forming stronger complexes with most metal cations, but which is only relevant at pH > 7, because DTPA is also a weaker acid [22].

Extractions with DTPA, buffered with tri-ethanolamine at pH 7.3, have been already used in 1976 to monitor plant-available Zn, Fe, Mn, and Cu concentrations in Colorado soils. After rapid extraction with the first 5 min, the dissolution equilibrium had not been reached after 2 h, but there was a strong correlation with the amounts obtained after 30 min, thus shortening the extraction time [23,24].

Doubling the voltage used in the EUF method led to similar results; the EUF conditions thus do not seem to be critical [19]. Additionally, though the extrapolated time to reach zero release differed between the models to some extent, the corresponding releasable concentrations calculated from the cumulative curves were rather similar, regardless of the kinetic model used.

From the metalliferous soil samples used, uptake of Zn, Li, and Sr could be well correlated with parameters of kinetic models from the release in 0.002 M DTPA obtained via the EUF method, while this was not the case for As, Cd, and leafy Cu (Table 4). In cases in which the desorption from the soil matrix to the soil solution is slower than the uptake by plant roots, the kinetics of soil desorption directly reflect the soil–plant transfer. This assumes that plant uptake is faster than dissolution [25,26]. If the plant uptake does not correspond with kinetic parameters, this means either that the plant does not take just what is released, but what it needs or rejects, or that the use of another electrolyte closer to physiological conditions might be more suitable. Surprisingly, the significance of correlations between plant uptake and kinetic parameters was similar, irrespective of the kinetic model used. Fitting all four parameters versus plant uptake into one equation by partial correlation would be more conclusive, but this feature was not contained in the statistics program which the author could use.

## 5. Conclusions

Most plant physiologists [1,2] assume that plant uptake of nutrients and metals is governed by respective amounts released into the soil solution. The This implicates that dissolution is rate determining but not the needs and the uptake mechanisms of the plant itself. Thus, plant-available fractions in soils were defined via selective dissolution obtained by various extractants, reaching some equilibrium by shaking. However, because plant uptake is a kinetic process, the specific point of this pilot study was to investigate the soil-to-plant transfer via the study of dissolution kinetics rather than a static process, as in the EUF procedure, the backward reaction is prevented by the removal of released items in the electric field, and the forward reactions become accessible.

Within this pilot study, only four types of soil could be used, but the range of concentrations and availabilities was amplified by adding PK fertiliser or a Cd-Ni-Pb solution to each of them. As a result, the uptake of Zn, Li, and also Ni and Pb into salad plants, and for roots also Cu, yielded significant correlations with kinetic dissolution parameters, whereas Cd and As did not. Thus, the plant does not necessarily absorb all that is released from the soil. Best-fit comparisons show that the optimum fit was obtained due to the released element but not due to the type of soil used (Table 4).

This should encourage further studies to use the EUF technique for other electrolytes and test plants, in order to optimise availability predictions, and on the other hand, to find nutrients and trace elements in combination with crops, the uptake of which does not depend merely on solubility. The method is quick—within half an hour, five solutions were obtained, and evaluations of the ICP–OES data might be programmed to obtain automatisation for routine use.

## Figures and Tables

**Table 1:** Test soils prior to the pot experiment.

Location	pH	Sand %	Silt %	Clay %	C-org mg/kg	Pb mg/kg	Ni mg/kg	Cd mg/kg	Cu mg/kg	Cr mg/kg	As mg/kg
Rabenstein	6.9	46	41	13	2.8	340	42	1.8	47	27	19
Arzwaldgraben	7.4	26	64	10	5.4	800	51	2.6	45	75	12
Zeltweg	6.2	30	62	8	4.1	130	43	0.24	44	58	35
Kraubath	7.3	44	47	9	4.6	21	24	<0.2	36	26	8

**Table 2:** Modelling the released concentrations from accumulated data.

Kinetic Model.	f(x)	Release Rate = dy/dt
Linear equation	y = b + at	dy/dt = a
Elovich equation	y = b + a ln(t)	dy/dt = a/t
Weber–Morris equation	y = b + a √t	dy/dt = a/√t
Power equation	ln(y) = b + a ln (t)	dy/dt = a/t·(b + a ln (t))

y = desorbed number of concentrations; t = time.

**Table 3:** Released amounts after 5, 10, 15, 20, and 25 min of electro-ultrafiltration in 0.002 M DTPA.

Al	As	Be	Cd	Co	Cr	Cu	Fe	Li	Mn	Ni	Pb	Sb	Sr	Ti	V	Zn
Rabenstein																
5.42	0.349	0.092	0.311	0.364	0.163	3.35	25.57	0.0466	36.68	1.34	21.62	0.121	0.912	0.082	0.096	17.34
9.93	0.232	0.078	0.203	0.588	0.136	2.01	36.24	0.0298	45.72	1.29	23.83	0.118	0.847	0.078	0.056	14.62
11.97	0.141	0.072	0.120	0.652	0.114	0.80	34.05	0.0194	41.16	0.75	18.44	0.108	0.641	0.098	0.048	9.03
14.37	0.102	0.074	0.085	0.711	0.117	0.47	34.06	0.0148	39.93	0.69	16.56	0.111	0.544	0.090	0.044	6.53
15.72	0.078	0.073	0.061	0.701	0.114	0.18	32.67	0.0119	37.13	0.53	13.67	0.110	0.475	0.096	0.042	4.87
Rabenstein + PK																
3.58	0.253	0.074	0.222	0.271	0.063	0.491	19.35	0.0271	31.54	0.89	18.69	0.112	0.865	0.009	0.027	14.93
7.44	0.194	0.076	0.168	0.471	0.079	0.327	29.44	0.0238	39.28	0.64	21.46	0.115	0.834	0.033	0.050	13.36
9.97	0.132	0.074	0.112	0.593	0.083	0.017	31.15	0.0176	39.20	0.50	19.10	0.112	0.668	0.054	0.039	9.35
11.70	0.095	0.073	0.076	0.642	0.083	0.016	30.45	0.0131	37.46	0.40	16.49	0.110	0.558	0.065	0.033	6.63
12.91	0.069	0.073	0.053	0.638	0.083	0.016	29.53	0.0104	35.34	0.30	13.69	0.109	0.477	0.069	0.030	4.91
Rabenstein + metal																
4.11	0.736	0.084	0.704	0.306	0.099	1.01	26.75	0.0275	29.16	42.75	18.93	0.116	0.910	0.045	0.037	14.73
8.62	0.560	0.076	0.529	0.535	0.105	0.88	39.97	0.0143	36.24	42.04	19.81	0.115	0.844	0.044	0.031	12.96
11.23	0.359	0.074	0.331	0.647	0.104	0.47	40.15	0.0110	35.47	29.27	17.33	0.112	0.813	0.061	0.035	8.63
13.04	0.239	0.073	0.219	0.685	0.108	0.15	38.57	0.0084	33.67	20.02	14.27	0.110	0.576	0.087	0.030	5.93
13.85	0.167	0.072	0.147	0.654	0.110	0.02	35.61	0.0068	30.88	13.79	11.56	0.108	0.480	0.080	0.029	4.26
Arzwaldgraben																
2.503	0.425	0.074	0.390	0.258	0.117	1.102	22.36	0.041	31.32	0.790	64.21	0.108	1.712	0.016	0.064	21.16
2.964	0.326	0.068	0.295	0.469	0.118	1.127	34.48	0.032	44.01	0.780	74.43	0.105	1.535	0.013	0.079	18.48
3.306	0.213	0.064	0.189	0.625	0.111	0.896	35.78	0.023	45.57	0.525	61.28	0.099	1.208	0.021	0.079	13.20
3.911	0.153	0.063	0.132	0.696	0.112	0.380	36.54	0.017	45.09	0.433	51.20	0.098	1.042	0.033	0.080	10.48
4.475	0.110	0.060	0.092	0.663	0.108	0.233	34.97	0.014	39.71	0.370	40.80	0.093	0.893	0.042	0.076	7.86
Arzwaldgraben + PK																
3.942	0.378	0.078	0.343	0.240	0.100	1.567	24.09	0.045	23.48	0.658	61.41	0.112	1.547	0.018	0.098	18.87
4.968	0.293	0.069	0.263	0.521	0.098	1.509	35.06	0.033	42.75	0.739	68.02	0.106	1.341	0.063	0.105	15.93
5.560	0.199	0.065	0.175	0.736	0.098	0.903	37.46	0.023	49.80	0.592	56.00	0.100	1.091	0.054	0.104	12.13
6.309	0.143	0.063	0.124	0.802	0.103	0.541	37.76	0.018	49.14	0.523	45.30	0.097	0.917	0.071	0.102	9.03
7.177	0.110	0.061	0.093	0.768	0.106	0.342	37.51	0.014	44.26	0.458	37.14	0.094	0.807	0.085	0.096	7.03
Arzwaldgraben + metal																
2.786	1.542	0.072	1.393	0.224	0.096	1.454	20.28	0.040	28.15	54.28	63.18	0.108	1.477	0.005	0.076	15.26
3.795	1.147	0.068	1.021	0.468	0.111	1.612	32.51	0.032	39.69	57.51	71.83	0.106	1.390	0.006	0.105	14.05
4.303	0.730	0.064	0.696	0.652	0.106	1.119	35.41	0.022	42.84	42.88	59.46	0.100	1.012	0.023	0.096	10.26
4.842	0.491	0.062	0.463	0.714	0.106	0.627	35.57	0.016	41.78	32.79	47.85	0.096	0.848	0.031	0.084	7.46
5.512	0.351	0.061	0.327	0.694	0.108	0.354	36.43	0.013	39.27	24.99	38.84	0.094	0.745	0.037	0.081	5.73
Zeltweg																
11.63	0.078	0.084	0.058	0.119	0.119	1.901	27.36	0.045	9.57	0.48	8.38	0.122	1.081	0.168	0.235	1.020
18.20	0.038	0.073	0.022	0.183	0.106	1.692	38.68	0.034	14.52	0.81	7.95	0.119	0.949	0.255	0.299	0.991
20.80	0.024	0.072	0.009	0.214	0.111	1.115	39.90	0.027	13.78	0.68	5.87	0.116	0.781	0.298	0.284	0.598
22.68	0.016	0.072	0.003	0.219	0.114	0.780	39.33	0.021	12.47	0.62	4.46	0.117	0.644	0.319	0.261	0.418
23.18	0.013	0.071	0.000	0.206	0.113	0.503	36.87	0.017	11.03	0.64	3.35	0.113	0.527	0.316	0.226	0.354
Zeltweg + PK																
11.91	0.096	0.091	0.075	0.150	0.147	1.702	32.84	0.040	15.88	0.48	9.34	0.121	1.053	0.192	0.222	0.752
18.08	0.037	0.074	0.020	0.147	0.108	1.427	42.61	0.023	13.92	0.39	8.41	0.115	0.895	0.262	0.253	0.259
21.29	0.023	0.074	0.007	0.171	0.115	0.995	43.17	0.019	12.14	0.28	6.43	0.115	0.750	0.310	0.248	0.106
22.74	0.017	0.073	0.001	0.176	0.114	0.594	40.76	0.015	10.71	0.20	4.83	0.113	0.614	0.319	0.233	0.003
23.12	0.013	0.072	0.000	0.166	0.117	0.377	38.00	0.013	9.54	0.15	3.62	0.110	0.517	0.316	0.204	0.043
Zeltweg + metal																
10.42	0.288	0.075	0.252	0.090	0.110	1.264	28.23	0.027	12.26	13.43	8.67	0.117	1.059	0.160	0.227	0.723
16.70	0.201	0.077	0.174	0.148	0.136	1.112	41.51	0.026	13.42	12.72	8.27	0.119	0.939	0.285	0.322	0.464
19.60	0.125	0.077	0.103	0.170	0.135	0.664	42.84	0.021	11.85	8.98	6.25	0.118	0.785	0.349	0.301	0.238
21.22	0.082	0.077	0.062	0.172	0.136	0.295	41.34	0.017	10.47	6.25	4.60	0.116	0.641	0.362	0.264	0.111
22.24	0.055	0.076	0.036	0.164	0.132	0.077	39.12	0.014	9.42	4.45	3.50	0.115	0.537	0.358	0.232	0.071
Kraubath																
6.74	0.070	0.079	0.050	0.100	0.116	0.599	24.47	0.023	10.07	1.04	2.21	0.118	1.336	0.135	0.120	2.24
8.74	0.030	0.063	0.014	0.087	0.092	0.562	34.81	0.009	9.92	0.39	1.33	0.108	1.073	0.170	0.133	1.80
9.72	0.021	0.061	0.007	0.096	0.095	0.448	36.57	0.007	8.94	0.32	1.03	0.104	0.829	0.214	0.128	1.27
10.73	0.043	0.059	0.029	0.103	0.100	0.408	37.05	0.006	8.34	0.35	0.89	0.101	0.693	0.247	0.122	1.05
11.56	0.044	0.058	0.030	0.107	0.099	0.337	36.48	0.005	7.76	0.40	0.83	0.098	0.573	0.259	0.111	0.85
Kraubath + metal																
3.88	0.903	0.063	0.817	0.226	0.096	1.933	21.81	0.045	23.28	23.38	57.70	0.114	1.833	0.020	0.070	14.03
4.23	0.688	0.059	0.652	0.497	0.096	2.302	30.36	0.036	41.72	28.38	67.22	0.108	1.508	0.010	0.089	12.61
4.75	0.445	0.057	0.416	0.688	0.101	1.509	32.94	0.025	46.16	22.74	55.35	0.103	1.164	0.027	0.091	9.03
5.41	0.283	0.054	0.260	0.744	0.101	1.189	32.62	0.017	44.99	16.99	42.86	0.097	0.907	0.031	0.082	6.40
5.85	0.226	0.053	0.207	0.729	0.101	0.966	32.70	0.014	41.76	14.50	37.52	0.097	0.828	0.040	0.078	5.41

PK: mineral fertiliser added; metal: metal solution added.

**Table 4:** Correlation coefficients between the contents in cropped lettuce and the kinetic parameters obtained from modelling the EUF data.

	Time to Reach Max. Conc.			Slope a			Intercept b			Maximum Releasable Concentrations		
Correlations	Leaf µg	Root µg	Sum µg	Leaf µg	Root µg	Sum µg	Leaf µg	Root µg	Sum µg	Leaf µg	Root µg	Sum µg
As												
Elovich	0.2134	0.0723	0.2523	0.2792	−0.3769	0.1625	−0.2916	0.3870	−0.1719	0.2799	−0.3881	0.1595
Weber–Morris	0.1848	0.0941	0.2289	0.2796	−0.3777	0.1626	−0.3054	0.3991	−0.1820	0.2778	−0.3930	0.1558
Linear	0.1642	0.0968	0.2078	0.2800	−0.3782	0.1629	0.2619	−0.3601	0.1503	0.2748	−0.4001	0.1503
Cd												
Elovich	−0.4708	−0.2838	−0.4652	0.2163	0.4371	0.2505	−0.2018	−0.4061	−0.2334	0.2095	0.4299	0.2433
Weber–Morris	−0.4370	−0.2290	−0.4277	0.2086	0.4151	0.2407	−0.1744	−0.3441	−0.2009	0.2012	0.4135	0.2338
Linear	−0.4465	−0.2328	−0.4368	0.2155	0.4370	0.2497	0.2339	0.4721	0.2707	0.1996	0.4253	0.2337
Cu												
Elovich	0.2296	−0.6832 *	0.0922	0.1437	−0.2802	0.0937	0.0058	0.4202	0.1092	0.2578	−0.2435	0.2314
Weber–Morris	0.2982	−0.8266 **	0.1345	0.1643	−0.3121	0.1091	−0.4483	0.7995 **	−0.3105	0.1800	−0.4092	0.1031
Linear	0.3102	−0.8229 **	0.1489	0.1746	−0.3290	0.1166	−0.3638	0.6371 *	−0.2548	0.1834	−0.4164	0.1051
Ni												
Elovich	−0.2415	−0.2803	−0.2559	0.7272 *	0.8545 **	0.7729 **	−0.7412 **	−0.8431 **	−0.7819 **	0.7274 *	0.8492 **	0.7719 **
Weber–Morris	−0.2074	−0.2984	−0.2319	0.7276 *	0.8541 **	0.7731 **	−0.7463 **	−0.8324 **	−0.7838 **	0.7341 *	0.8499 **	0.7775 **
Linear	−0.0675	−0.1540	−0.0874	0.7279 *	0.8537 **	0.7733 **	0.6245 *	0.8393 **	0.6858 *	0.7356 **	0.8491 **	0.7786 **
Pb												
Elovich	0.3787	0.4582	0.4743	0.6204 *	**0.5133**	0.6808 *	**−0.6275**	**−0.5196**	−0.6888 *	0.6314 *	**0.5337**	0.6975 *
Weber–Morris	0.4863	0.4937	**0.5707**	0.6208 *	**0.5140**	0.6814 *	−0.6314 *	**−0.5238**	−0.6935 *	0.6329 *	**0.5351**	0.6992 *
Linear	0.5485	0.5084	0.6240 *	0.6212 *	**0.5146**	0.6819 *	**0.5309**	**0.4306**	**0.5791**	0.6338 *	**0.5357**	0.7001 *
Zn												
Elovich	0.3927	0.1565	0.3615	0.8328 **	0.7785 **	0.8488 **	−0.2999	−0.2757	−0.3048	0.7588 **	0.7069 *	0.7729 **
Weber–Morris	0.4729	0.3029	0.4564	0.8316 **	0.7770 **	0.8475 **	−0.7877 **	−0.6916 *	−0.7946 **	0.7996 **	0.7584 **	0.8170 **
Linear	0.5930	0.3597	**0.5685**	0.8304 **	0.7755 **	0.8463 **	0.8579 **	0.8648 **	0.8860 **	0.8155 **	0.7575 **	0.8303 **
Li	Li											
Elovich	−0.1197	−0.3346	−0.2006	**0.5613**	0.4747	0.6433 *	**−0.5397**	−0.4980	−0.6299 *	**0.5900**	0.4108	0.6523 *
Weber–Morris	0.0549	−0.0205	0.0447	**0.5640**	0.4757	0.6461 *	**0.5165**	−0.3511	0.3780	0.6633 *	0.2855	0.6854 *
Linear	−0.3865	−0.4020	−0.4634	**0.5511**	0.4694	0.6325 *	0.0585	−0.3236	−0.0343	0.3898	−0.0718	0.3377
Sr	Sr											
Elovich	−0.0994	0.0188	−0.0920	0.0443	0.3374	0.0709	−0.3794	−0.4329	−0.3955	−0.0200	0.2541	0.0048
Weber–Morris	0.4391	0.6361 *	0.4694	0.3255	0.3134	0.3344	**−0.5136**	−0.4951	**−0.5276**	0.4156	0.4522	0.4313
Linear	0.6141 *	**0.5901**	0.6306 *	0.2541	0.3038	0.2661	−0.1086	−0.0917	−0.1104	0.3961	0.4504	0.4133

Parameters a and b refer to the respective definitions given in Table 2. * means *p* < 0.05 or >95% of confidence, ** means *p* < 0.01 or >99% of confidence. Correlation coefficients > 0.5 have been marked bold.

**Table 5:** Probability values between the contents in cropped lettuce and the kinetic parameters obtained from modelling the EUF data.

	Time to Reach Max. Conc.			Slope a			Intercept b			Maximum Releasable Concentrations		
Probability	Leaf µg	Root µg	Sum µg	Leaf µg	Root µg	Sum µg	Leaf µg	Root µg	Sum µg	Leaf µg	Root µg	Sum µg
As												
Elovich	0.501	0.288	0.293	0.406	0.253	0.633	0.384	0.24	0.613	0.403	0.238	0.638
Weber–Morris	0.513	0.301	0.307	0.405	0.252	0.633	0.361	0.224	0.592	0.409	0.231	0.649
Linear	0.501	0.299	0.297	0.404	0.251	0.632	0.437	0.277	0.659	0.413	0.224	0.658
Cd												
Elovich	0.144	0.398	0.149	0.523	0.179	0.457	0.552	0.216	0.489	0.539	0.187	0.472
Weber–Morris	0.179	0.498	0.189	0.538	0.205	0.475	0.608	0.300	0.553	0.553	0.206	0.488
Linear	0.169	0.491	0.179	0.524	0.179	0.458	0.489	0.143	0.420	0.554	0.191	0.486
Cu												
Elovich	0.497	0.020 *	0.788	0.673	0.404	0.782	0.987	0.198	0.749	0.444	0.470	0.493
Weber–Morris	0.373	0.002 **	0.693	0.629	0.350	0.749	0.167	0.003 **	0.352	0.597	0.211	0.763
Linear	0.353	0.002 **	0.662	0.608	0.323	0.733	0.271	0.035 *	0.449	0.589	0.203	0.758
Ni												
Elovich	0.474	0.404	0.448	0.011 *	0.001 **	0.005 **	0.009 **	0.001 **	0.004 **	0.011 *	0.001 **	0.005 **
Weber–Morris	0.541	0.373	0.498	0.011 *	0.001 **	0.005 **	0.008 **	0.001 **	0.004 **	0.010 *	0.001 **	0.005 **
Linear	0.844	0.651	0.798	0.011 *	0.001 **	0.005 **	0.040 *	0.001 *	0.020 *	0.010 **	0.001 **	0.005 **
Pb												
Elovich	0.251	0.156	0.140	0.042 *	0.106	0.021 *	0.039 *	0.101	0.019 *	0.037 *	0.091	0.017 *
Weber–Morris	0.129	0.123	0.067	0.042 *	0.106	0.021 *	0.037 *	0.098	0.018 *	0.037 *	0.090	0.017 *
Linear	0.081	0.110	0.040 *	0.041 *	0.105	0.021 *	0.093	0.186	0.062	0.036 *	0.089	0.016 *
Zn												
Elovich	0.231	0.647	0.273	0.001 **	0.005 **	0.001 **	0.374	0.416	0.361	0.007 **	0.014 *	0.005 **
Weber–Morris	0.141	0.364	0.157	0.001 **	0.005 **	0.001 **	0.004 **	0.018 *	0.003 **	0.003 **	0.007 **	0.002 **
Linear	0.054	0.275	0.067	0.002 **	0.005 **	0.001 **	0.001 **	0.001 **	0.000 **	0.002 **	0.007 **	0.002 **
Li												
Elovich	0.722	0.316	0.555	0.073	0.139	0.033 *	0.087	0.119	0.038	0.054	0.183	0.027 *
Weber–Morris	0.874	0.956	0.894	0.071	0.137	0.032 *	0.103	0.298	0.250	0.029 *	0.360	0.021 *
Linear	0.238	0.220	0.151	0.079	0.144	0.037 *	0.860	0.329	0.922	0.229	0.867	0.297
Sr												
Elovich	0.695	0.749	0.730	0.897	0.339	0.838	0.250	0.190	0.229	0.954	0.451	0.991
Weber–Morris	0.177	0.032 *	0.145	0.329	0.350	0.315	0.106	0.121	0.096	0.203	0.163	0.185
Linear	0.045 *	0.057	0.037	0.452	0.374	0.429	0.750	0.774	0.746	0.229	0.165	0.207

Parameters a and b refer to the respective definitions given in Table 2. * means *p* < 0.05 or >95% of confidence, ** means *p* < 0.01 or >99% of confidence.

## Data Availability

Additional data available from the author upon request.
